# Glutamine Synthetase Is a Genetic Determinant of Cell Type–Specific Glutamine Independence in Breast Epithelia

**DOI:** 10.1371/journal.pgen.1002229

**Published:** 2011-08-11

**Authors:** Hsiu-Ni Kung, Jeffrey R. Marks, Jen-Tsan Chi

**Affiliations:** 1 Duke Institute for Genome Sciences and Policy, Duke University Medical Center, Durham, North Carolina, United States of America; 2 Department of Molecular Genetics and Microbiology, Duke University Medical Center, Durham, North Carolina, United States of America; 3 Department of Anatomy and Cell Biology, School of Medicine, National Taiwan University, Taipei, Taiwan; 4 Department of Surgery, Duke University Medical Center, Durham, North Carolina, United States of America; Massachusetts Institute of Technology, United States of America

## Abstract

Although significant variations in the metabolic profiles exist among different cells, little is understood in terms of genetic regulations of such cell type–specific metabolic phenotypes and nutrient requirements. While many cancer cells depend on exogenous glutamine for survival to justify the therapeutic targeting of glutamine metabolism, the mechanisms of glutamine dependence and likely response and resistance of such glutamine-targeting strategies among cancers are largely unknown. In this study, we have found a systematic variation in the glutamine dependence among breast tumor subtypes associated with mammary differentiation: basal- but not luminal-type breast cells are more glutamine-dependent and may be susceptible to glutamine-targeting therapeutics. Glutamine independence of luminal-type cells is associated mechanistically with lineage-specific expression of glutamine synthetase (GS). Luminal cells can also rescue basal cells in co-culture without glutamine, indicating a potential for glutamine symbiosis within breast ducts. The luminal-specific expression of GS is directly induced by *GATA3* and represses glutaminase expression. Such distinct glutamine dependency and metabolic symbiosis is coupled with the acquisition of the GS and glutamine independence during the mammary differentiation program. Understanding the genetic circuitry governing distinct metabolic patterns is relevant to many symbiotic relationships among different cells and organisms. In addition, the ability of GS to predict patterns of glutamine metabolism and dependency among tumors is also crucial in the rational design and application of glutamine and other metabolic pathway targeted therapies.

## Author Summary

Different types of cells have distinct ways of utilizing nutrients and generating energy, thus resulting in distinct nutrient needs. Such cell type–specific metabolic differences are associated with many biological processes and force the symbiosis between different cells and organisms. For example, glutamine symbiosis is a well-recognized phenomenon due to different glutamine synthesis ability. In human cancers, glutamine is also recognized as an important and essential nutrient, termed glutamine addiction. But very little is known about how glutamine addiction varies among different tumors of diverse cellular origins, which hinders personalized therapeutic strategies. Here, we found that basal-type breast cancer cells were sensitive to glutamine deprivation while luminal-type breast cancer cells were not. Luminal cell–specific glutamine independence results from expression of glutamine synthetase conferring the ability to synthesize glutamine. Glutamine synthetase also represses glutaminase and contributes to the maintenance of the polarized expression of glutamine synthetase and glutaminase among breast cancer cells. Collectively, these data illustrate cross-talk between mammary differentiation programs and unique nutrient requirements, which may offer novel therapeutics for basal-type breast cancers.

## Introduction

There are a large number of differentiated cell types in the human body. Even among the cells collectively known as fibroblasts [1], endothelial [2] and smooth muscle cells [3], gene expression analysis has identified an unexpected level of positional memory and topographic differentiation. Such functional specialization contributes to the phenotypic variations of many human diseases, including cancer. For example, gene expression analysis of breast cancers has identified five intrinsic subtypes (luminal A, luminal B, basal, HER2+, and normal-like) with unique clinical and histological properties [4], [5]. The classification nomenclature is based on the putative progenitor cell(s) for breast carcinogenesis with properties consistent with derivation from the basal and luminal epithelia arrested at specific differentiation stages or from different mature epithelial cells [4]–[7]. Importantly, these subtype-specific gene expression and phenotypic variations are also observed in many breast cancer cell lines with similar molecular phenotypes [8]–[11]. A number of studies have isolated the different populations of primary epithelial cells to investigate their relevant cellular origins and metabolic features for different breast cancer types [7], [12], [13]. Although the cellular origin of luminal and basal-like breast tumor has not been resolved [14], [15], cell lineage still appears to confer an important source of patterned heterogeneity to the disease.

Although gene expression analysis has yielded important insights into the cellular differentiation and various properties associated with tumors from different cell types, very little is known about the corresponding metabolic phenotypes and nutrient requirements. The processes of oncogenic transformation place energy demands on cancer cells to support proliferation, expansion, and invasion. Dysregulated tumor metabolism is a critical part of oncogenesis and may be targeted for therapeutic benefits [16], [17]. One prominent example of dysregulated tumor metabolism is “aerobic glycolysis” as recognized by Otto Warburg [18]. Most normal mammalian cells shift to glycolysis for energy generation when oxygen is inadequate for effective oxidative phosphorylation under hypoxia. But tumor cells tend to favor glycolysis even with the availability of oxygen, hence termed “aerobic glycolysis” [19]. Such preferential use of glycolysis leads to vigorous glucose uptake and explains the ability of the tracer glucose analog Fluorine-18 (F-18) FDG to image human cancers in FDG-PET. Such understanding of altered metabolism and nutrient requirement in cancer cells may allow us to exploit these differences for diagnostic and therapeutic benefits.

Another aspect of dysregulated tumor metabolism is manifested as altered requirements for amino acids. For example, patients with acute lymphocytic leukemia (ALL) benefit from asparaginase treatment as the leukemic cells require large amounts of exogenous asparagine due to a deficiency in this metabolic pathway [20]. Recently, evidence is also accumulating for the essential role of glutamine for cancer cells as a building block for protein synthesis, to supply cellular ATP, as a metabolic intermediate for nucleotide synthesis, and for its anti-oxidative capacity [21], [22]. Such glutamine dependence or addiction is reflected in the growth restriction and cell death in glutamine limiting conditions. The glutamine addiction is also critical for c-myc-mediated oncogenesis [23]–[25], linked with glucose requirement [26], and proposed as an attractive target for therapeutic intervention [22], [27].

The catabolism of glutamine is initiated by glutaminolysis mediated by two different subtypes of mitochondrial glutaminase (kidney or liver-type encoded by *GLS* or *GLS2* respectively) to become glutamate [28]. The intracellular pool of glutamate is a versatile metabolic intermediate that connects with a wide variety of distinct biological processes including synthesis of the anti-oxidant glutathione, amino acid catabolism through transamination, and conversion to α-ketoglutarate as a substrate for the TCA cycle. This process of glutaminolysis by glutaminases has been shown to mediate signaling events [29], to be coupled with c-myc oncogenesis [25], and proposed as a critical step in targeting glutamine metabolism [24], [27]. In some cell types, glutamine can be generated from intracellular glutamate through glutamine synthetase (GS, encoded by *GLUL*, glutamate-ammonia ligase) catalyzing the reverse reaction of the glutaminases. This process is important for removal of ammonia or glutamate depending on the cellular context [30]. While glutaminase is known as an important regulator of glutamine requirement, few studies have focused on glutamine synthetase as a potential determinant of glutamine requirement. Although normal glutamine metabolism is well understood, the genetic parameters and mechanisms of variation in this key nutrient pathway among tumors are largely unknown.

Deprivation of glutamine and other amino acids triggers a canonical amino acid response (AAR) in most mammalian cells that is measurable by gene expression changes [31]. The free and uncharged t-RNA associated with glutamine deprivation activates a serine/threonine-protein kinase *GCN2* which phosphorylates eIF2α and inhibits cap-dependent translation [32]. While reducing the global translation rate, eIF2α phosphorylation also preferentially increases the translation of ATF4 and other mRNAs [31]. The increased level of ATF4 protein triggers the AAR gene expression program characterized by the induction of *XBP1* and *DDIT3* as an adaptive response to amino acid deprivation. The importance of the AAR is demonstrated by the fact that deficiency of ATF4 compromises the AAR and renders cells susceptible to amino acid deprivation and oxidative stresses [33].

Through the analysis of how different breast cancer cells respond to glutamine deprivation, we have found a dramatic difference in the glutamine requirement among different breast cancer cells which tracks with the luminal versus basal type. These metabolic differences can be explained by cell-type specific expression of glutamine-metabolizing genes and enzymes likely acting in concert with cell type specific oncogenic programs. Therefore, we have provided a series of fundamental building blocks to understand how differentiation is coupled with distinct glutamine utilization in normal and neoplastic breast epithelia. Such an understanding will be relevant to both the mechanistic understanding of metabolic phenotypes and present insights into how best to select subsets of breast cancer patients most likely to benefit from glutamine-targeting therapies.

## Results

### Breast cancer cells exhibit subtype-specific phenotype of glutamine dependence

**Figure 1:**
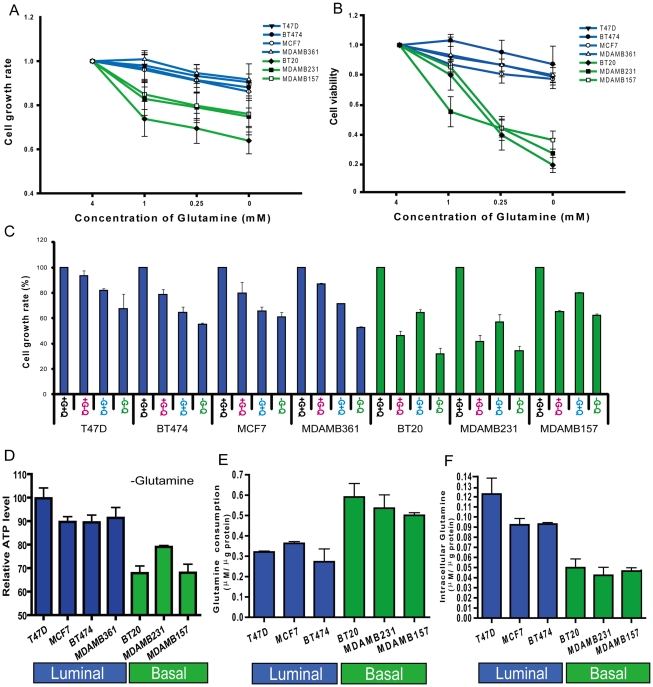
Glutamine addiction phenotypes among different breast cancer cell lines. (A, B) The normalized cell growth (MTT assay) (A) and viability (trypan blue exclusion assay) (B) of seven indicated breast cancer cell lines (luminal-type: blue, basal-type: green) at different glutamine concentrations. (C) The normalized cell growth of the seven indicated cell lines under control (+G+Q), glutamine deficient (+G-Q), glucose deficient (-G+Q) and glucose/glutamine deficient condition (-G-Q). (D) The percentage of reduction in normalized cellular ATP of the indicated cell lines when cultured in glutamine deficient media for the indicated breast cancer cell lines. (E, F) The glutamine consumption (E) and the intracellular glutamine concentration (F) of the indicated breast cancer cell lines grown in regular media.

Many cancer cells require glutamine for survival and proliferation and thus exhibit a phenotype of “glutamine dependence” or “addiction” [22]. To determine whether such phenotypes could be also found in breast cancer cells, we tested how glutamine deprivation affected seven different breast cancer cell lines. Consistent with the idea of glutamine dependence, three cell lines (BT20, MDAMB231, and MDAMB157) had significantly reduced growth (MTT assay, Figure 1A) and prominent cell death (trypan blue exclusion assay, Figure 1B) upon glutamine deprivation for 48 h. Unexpectedly, glutamine deprivation had only modest effects on the growth and viability (Figure 1A, 1B) of the other four cell lines (T47D, BT474, MCF7, and MDAMB361) indicating relative glutamine independence. When we examined the properties associated with the distinct need for glutamine, we found the cell lines that exhibit glutamine dependence are all of the basal-type whereas the four lines that are more glutamine independent are luminal-type cells (Figure 1A, 1B) [34].

As glucose and glutamine are two important energy sources for cancer cells we compared how deprivation of glutamine and glucose affected the growth of these breast cell lines. In the three basal-type cell lines, glutamine depletion had a stronger effect on cell growth than glucose depletion (Figure 1C). In contrast, glucose depletion had a more dramatic influence on cell growth than glutamine depletion in the four luminal cell lines (Figure 1C). These results suggested that there is a consistent variation in glutamine phenotype associated with cell lineage in breast cancers.

One important function of glutamine is to serve as an energy source in generating cellular ATP. To determine the relative importance of glutamine to ATP generation in the breast cancer cell lines, we measured ATP in cells grown in media containing either normal levels of glutamine (4mM) or no glutamine for 12 hours. Glutamine deprivation led to a much more significant reduction in ATP generation in the basal-type cells than the luminal-type breast cancer cell lines (Figure 1D). These results further support the concept that glutamine is a more important energy source in basal than luminal breast cell lines.

To further analyze glutamine metabolism among different cell types, we measured the consumption of glutamine in the medium and intracellular glutamine levels. When compared with luminal-type cells, the basal cell lines had significantly higher levels of glutamine consumption (Figure 1E) and lower intracellular glutamine concentrations (Figure 1F). Collectively, these data strongly support the concept of distinct glutamine metabolism and varying dependence for external glutamine between basal and luminal type breast cancer cells.

### Differential expression of glutamine-metabolizing enzymes in the basal and luminal breast cancer cell lines

**Figure 2:**
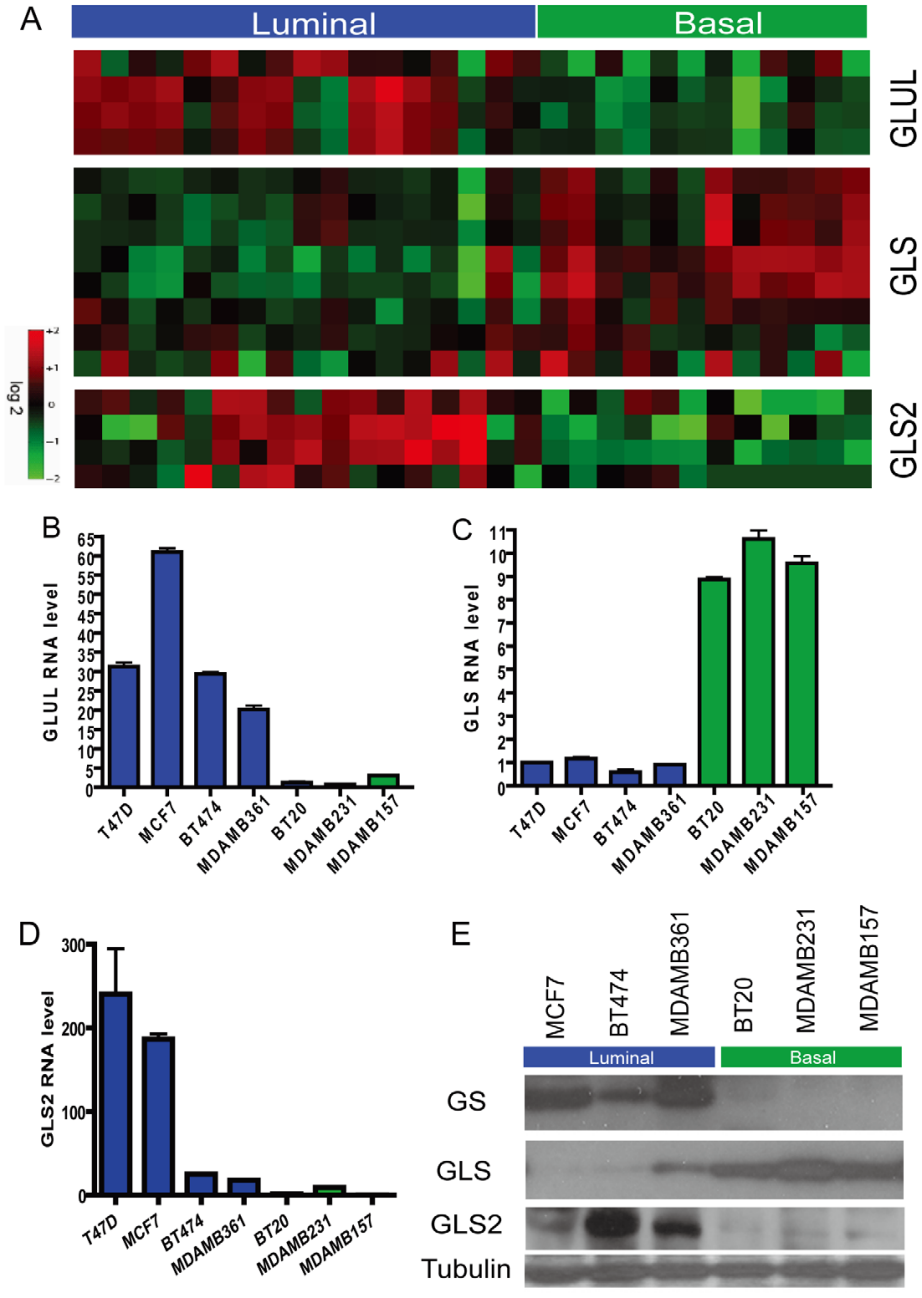
Differential expression of genes encoding glutamine-metabolizing enzymes in the basal and luminal breast cancer cell lines. (A) The heatmap showing the expression levels of probesets for *GLUL, GLS* and *GLS2* in the microarray data of indicated breast cancer cell lines known to be of luminal (blue) and basal (green) type. (B, C, D) The levels of mRNA expression of *GLUL* (B), *GLS* (C) and *GLS2* (D) of the indicated luminal and breast cell lines determined with real-time PCR. (E) The levels of protein products of *GLUL, GLS,* and *GLS2* in the indicated luminal and basal breast cell lines determined by Western blots.

We hypothesized that such distinct glutamine dependence among basal and luminal breast cancer cell lines may be caused by variable expression of key enzymes involved in glutamine metabolism. Glutamine synthetase (GS encoded by *GLUL* – glutamate-ammonia ligase) and glutaminase (*GLS* – kidney form or *GLS2* – liver form) mediate the opposite reaction in the reversible conversion between glutamate and glutamine. GS mediates the capture of an ammonia group by glutamate to synthesize glutamine, while glutaminase catalyzes the breakdown of glutamine to glutamate. We first examined the RNA expression of these genes in a microarray expression set [34] and found that the expression of *GLUL* (GS) was significantly higher in the luminal cell lines. In contrast, the expression of *GLS* (glutaminase, kidney) was higher in the basal lines. While lacking *GLS* expression, the luminal breast cell lines have a higher level of *GLS2* (Figure 2A). We confirmed this cell-type specific differential mRNA expression of *GLUL*, *GLS* and *GLS2* with real-time PCR (Figure 2B, 2C, and 2D). Differential expression was also found at the protein level as shown by the western blots for *GLUL* (GS) and *GLS2* (in luminal cells) and *GLS* (in basal cells) (Figure 2E).

### Differential expression of glutamine-metabolizing enzymes in primary human breast cancers

**Figure 3:**
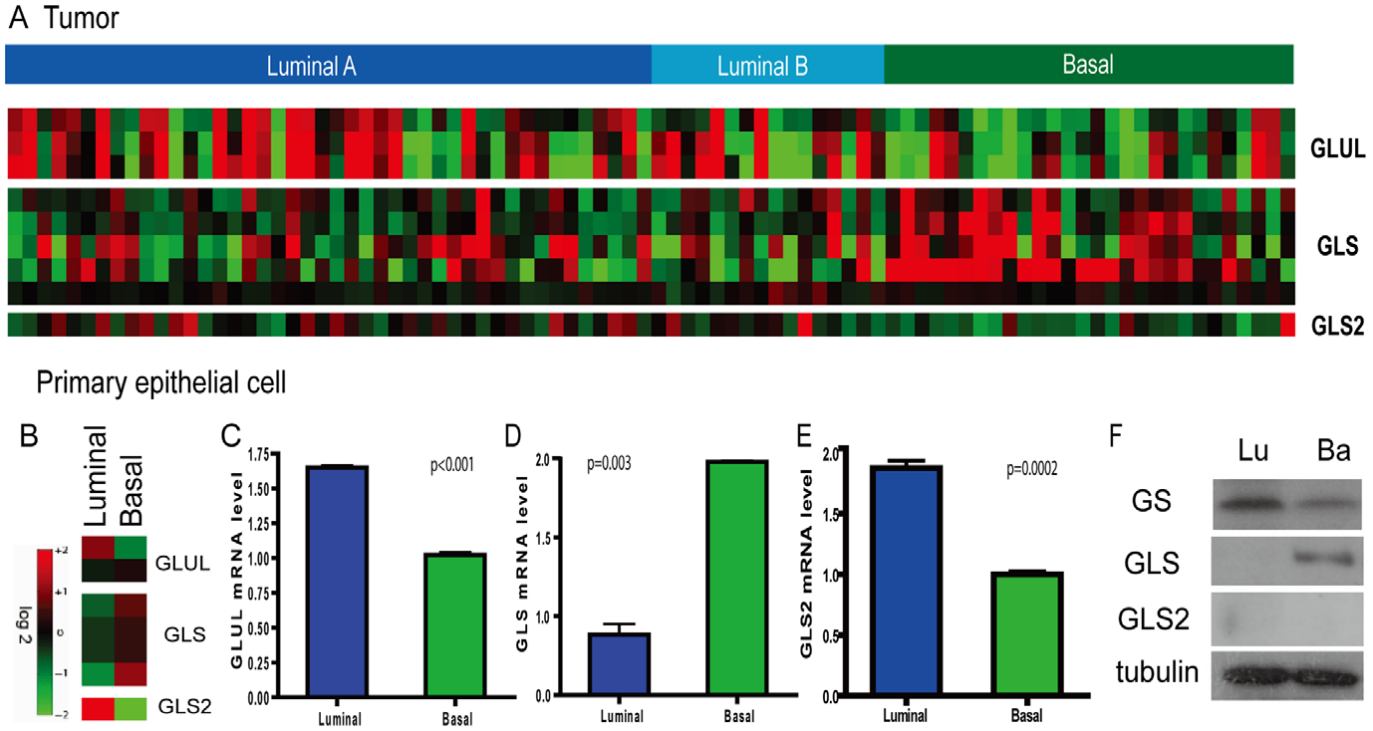
Persistent differential expression of genes encoding glutamine-metabolizing enzymes in the basal and luminal breast tumors and primary epithelial cells. (A) The average expression levels were shown for the, *GLS* and *GLS*2 in the luminal and basal breast tumors. (B) The heatmap showing the expression levels of probesets for *GLUL*, *GLS* and *GLS2* in the basal and luminal epithelial cells. (C, D, E) The expression levels determined by real-time PCR were shown for the *GLUL* (C), *GLS* (D), and GLS2 (E) in the primary luminal and basal breast epithelial cells. (F) The levels of protein products of *GLUL* and *GLS* in the basal and luminal breast epithelial cells by Western blots.

We next examined whether the expression patterns of *GLUL, GLS* and *GLS2* found in luminal and basal cell lines were also reflected in the respective subtypes of primary human breast cancers. In a breast tumor expression dataset [35], we found significantly different expression levels of *GLUL*, *GLS* and *GLS2* in the corresponding luminal (luminal A and B) and basal-types of breast tumors (Figure 3A). We also examined the expression levels of these three genes in the same dataset within the 5 intrinsic subtypes [35] and found significantly different expression between the luminal A and basal tumors (Figure S1). This concordance indicates the differential expression of *GLUL*, *GLS* and *GLS2* in the luminal and basal-type cancer cell lines reflects similar systematic differences in primary breast tumors.

### Lineage-specific expression of glutamine-metabolizing enzymes in primary breast epithelia

To determine whether differential expression of genes driving glutamine metabolism is an intrinsic cell-lineage phenomenon in the breast, we examined their expression levels in normal non-transformed basal and luminal epithelial cells. Primary luminal and basal breast epithelial cells were separated based on surface expression of EPCAM (TACSTD1) from reduction mammoplasty specimens and gene expression levels were determined by microarray analysis [12]. Analysis of isogenic basal and luminal epithelial cells showed that the mRNA levels of *GLUL, GLS* and *GLS2* exhibited similar cell-type specific expression in normal breast cells (Figure 3B). These findings were also confirmed by real-time PCR (Figure 3C–3E). In addition, expression of the GLUL (GS) and GLS proteins showed corresponding luminal and basal-specific expression patterns (Figure 3F). The level of GLS2 protein was below detection levels in both primary epithelial cells (Figure 3F). These results suggest that differential expression of glutamine metabolizing enzymes in cancers may be ascribed to systematic differences in cell lineage observed in normal basal and luminal epithelial cells.

### Luminal-specific GLUL expression as a determinant of glutamine independence

**Figure 4:**
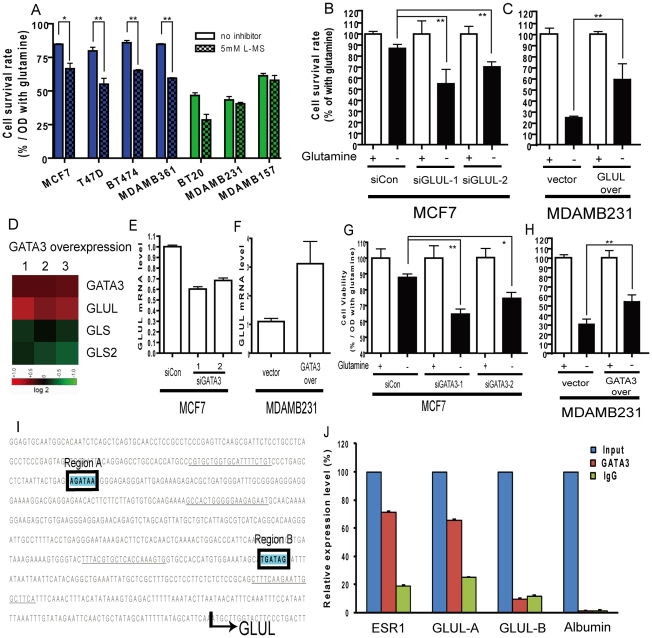
Contribution of luminal expression of GLUL and GATA3 to the glutamine-independence phenotype. (A) The normalized cell survival of breast cancer cell lines (luminal-type: blue, basal-type: green) with or without treatment with GS inhibitor, L-MS, in the absence of glutamine. (B) The degree of cellular survival under glutamine deprivation for MCF7 (luminal cell) treated with either control or two siRNAs targeting *GLUL*. (C) The degree of cellular survival under glutamine deprivation for MDAMB231 (basal cell) transfected with either control vector or *GLUL* overexpression construct. (D) The changes of *GLUL*, *GLS* and *GLS2* in the mouse mammary epithelia cells transfected with GATA3 from array analysis derived from an independent study [38]. (E) The levels of *GLUL* in MCF7 cells treated with either control or siRNA targeting GATA3. (F) The levels of *GLUL* in the MDAMB231 cells transfected with either control vectors or GATA3 expression constructs. (G) The relative survival under glutamine deprivation of MCF7 cells treated with control or two independent siRNAs targeting GATA3. (H) The cell survival rates shown in MDAMB231 cells with overexpression of control vector or GATA3 under glutamine deprivation. (I) The promoter regions of the *GLUL* with two potential binding sites of GATA3 are shown. (The sequences underlined indicate primer locations.) (J) The enrichment of different promoter regions of GLUL, ER (positive control) and albumin (negative control) which have been immunoprecipitated with GATA3 and control IgG antibodies. (All statistical comparisons: *p<0.05, **p<0.01)

Given the well-recognized glutamine dependency of many cancer cells, we investigated the roles of *GLUL* and *GLS2* in the relative glutamine *independence* of the luminal-type cells. We first treated cells with a GS inhibitor (L-MS [36]) for 48 h and measured cell viability under glutamine deprivation. We found that L-MS reduced the survival of the luminal cell lines but had no statistically significant effect over glutamine starvation on all three tested basal cell lines (Figure 4A) indicating that GS is involved in the glutamine independence of the luminal cells.

Next, we performed genetic experiments to examine the role of specific genes in the glutamine phenotype. Silencing of *GLUL* (encoding GS) in the luminal MCF7 line significantly reduced the RNA and protein expression of GS (Figure S2A and S2B) and led to a significant reduction in glutamine independence (Figure 4B). In contrast, similar silencing of *GLS2* did not affect survival under glutamine deprivation (Figure S3). In addition, the ectopic overexpression of *GLUL* (verified in Figure S4A, S4B) in the basal MDAMB231 cells conferred partial glutamine independence by significantly increasing the cell survival under glutamine deprivation (Figure 4C). Taken together, these data suggest that GS expression significantly contributes to the differential glutamine phenotypes observed in breast cancer cell lines.

### GATA3 regulates the luminal-specific expression of GS and glutamine independence

We next investigated potential regulatory mechanisms for the subtype-specific expression of glutamine metabolizing enzymes. During the differentiation of luminal epithelial cells, *GATA3* is an important master regulatory transcription factor [37], [38]. The expression of GATA3 in luminal and basal cells is systematically different as previously noted [11], [39]. Using real-time PCR, we also demonstrated the cell-type specific expression of GATA3 mRNA in MCF7 (luminal) and MDAMB231 (basal) cells (Figure S5A). Re-analysis of microarray data of the overexpression of *GATA3* in mouse breast epithelial cells [38] shows induction of *GLUL* and repression of *GLS* and *GLS2* (Figure 4D). These data suggested a role for the lineage factor *GATA3* in regulating the luminal and basal-specific expression of *GLUL* and *GLS*.

We directly tested the role of *GATA3* in regulating the glutamine phenotype in breast cancer cell lines. The mRNA and protein levels of GATA3 could be effectively reduced by gene silencing through siRNAs (Figure S5B, S5C). Silencing of *GATA3* in MCF7 cells led to significant reduction in *GLUL* at both the RNA and protein levels (Figure 4E, Figure S5C). Conversely, overexpression of *GATA3* in the basal MDAMB231 line (Figure S5D) led to a significant upregulation of *GLUL* (Figure 4F, Figure S5E). Furthermore, the silencing of *GATA3* in MCF7 cells reduced the survival under glutamine deprivation (Figure 4G, Figure S3), and overexpression of *GATA3* in MDAMB231 cells increased the resistance to glutamine deprivation (Figure 4H), consistent with a direct role for *GATA3* mediated *GLUL* expression in the glutamine independence of luminal breast cells. In addition, the glutamine independence caused by *GLUL* (Figure S6A) or *GATA3* (Figure S6B) overexpression in MDAMB231 cells was also abolished with treatment of L-MS (GS inhibitor), indicating of the importance of the catalytic activities of GS.

### GATA3 directly binds to the promoter of *GLUL*

Given the ability of *GATA3* to increase the expression of *GLUL*, we examined the promoter region of *GLUL* and found two potential *GATA3* binding sites at −524 to −518 bp (region A) and −200 to −194 bp (region B) upstream of the transcriptional start site (Figure 4I). We used chromatin immunoprecipitation (ChIP) to test whether *GLUL* may be a direct downstream target of *GATA3* transactivation. Consistent with previous data [40], the promoters of *ESR1* (estrogen receptor alpha), but not albumin, were enriched in the *GATA3* ChIP samples. Of the two putative *GATA3* binding sites in the *GLUL* promoter, the distal region A but not the more promoter proximal region B, was significantly enriched in the *GATA3* ChIP samples (Figure 4J) indicating that *GATA3* protein can directly bind to a regulatory region of *GLUL* suggesting that this gene is a target of the luminal transcription factor and further serving to explain the lineage specific requirement for glutamine.

### Cell type–specific transcriptional responses to glutamine deprivation

**Figure 5:**
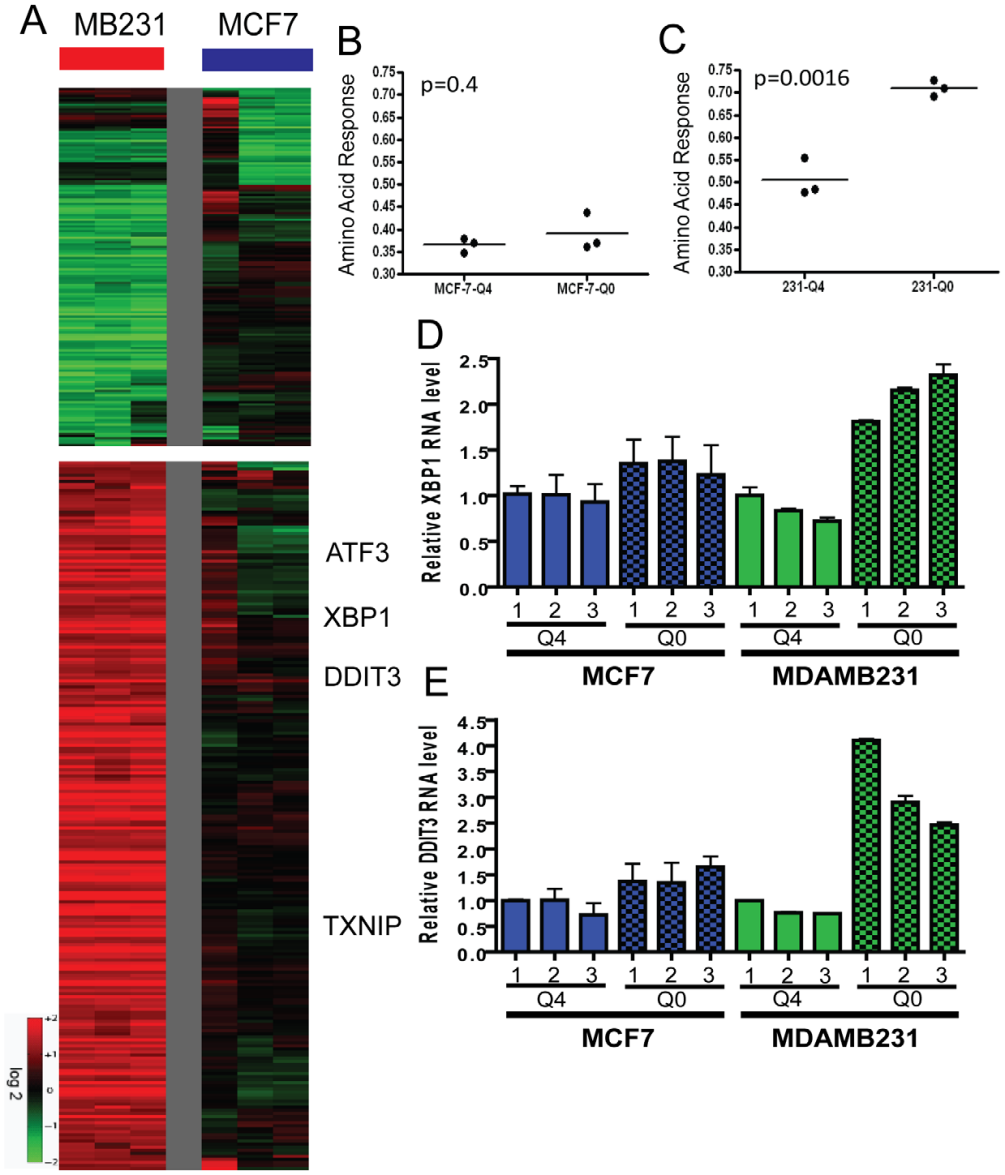
The transcriptional response of breast cancer cell lines to glutamine deprivation. (A) The heatmap representing the transcriptional response of MCF7 (luminal) and MDAMB231 (basal) cells to glutamine deprivation. (B, C). The predictive probability of the amino acid response (AAR) are shown for the luminal (B, p = 0.4, unpaired t-test) and basal (C, p = 0.0016) cancer cell lines grown under normal (4 mM, Q4) and glutamine-deficient (0 mM, Q0) medium. (D, E) The expression of selected canonical amino acid response genes including XBP1 (D) and DDIT3 (E) in MCF7 and MDAMB231 under indicated concentrations of glutamine with real time RT-PCR.

The deprivation of amino acids in mammalian cells leads to the stabilization of the *ATF4* (activating transcription factor 4) protein and resulting induction of a canonical gene expression program known as the amino acid response (AAR) [41]. The response includes the induction of *XBP1* (X-box binding protein 1) and *DDIT3* (DNA-damage-inducible transcript 3) which are essential for survival under amino acid deprivation [41]. Given the distinct growth and survival response of luminal and basal breast cells to glutamine deprivation, we used microarrays to compare their transcriptional responses on a global scale. Triplicate plates of MCF7 and MDAMB231 cells were cultured under both control (4 mM glutamine/Q4) and glutamine-depleted (no glutamine/Q0) conditions for 24 hours. RNA from each plate was interrogated with Affymetrix GeneChip U133-A2 arrays (results deposited in Gene Expression Omnibus (GSE26370)). Gene expression profiles of the 12 arrays were normalized by RMA and the transcriptional changes of glutamine deprivation in both cell types were derived by zero-transformation against the average expression levels of the control samples as performed previously [42]–[44]. Probes sets showing at least two fold changes in at least two samples (n = 405) were selected and arranged by hierarchical clustering according to similarities in expression patterns (Figure 5A). This analysis showed that glutamine deprivation induced a strong gene expression response in MDAMB231 (MB231) but less so in MCF7 cells (Figure 5A). We found that the canonical AAR genes were induced by glutamine deprivation only in MDAMB231 cells (Figure 5A). A previous study showed that glutamine deprivation inhibits cell growth by inducing the tumor suppressor gene TXNIP [29]. This gene was also induced only in the MDAMB231 line. We applied a published gene expression study of histidine deprivation [45] as training data and estimated the degree of AAR using a binary regression model. MDAMB231 but not the MCF7 line exhibited a significantly higher probability of AAR after glutamine deprivation using this approach (Figure 5B and 5C). The stronger amino acid response in the MDAMB231 cells was also confirmed by real-time PCR for *XBP1* (Figure 5D) and *DDIT3* (Figure 5E). These data provide further evidence that glutamine deprivation induces a much dramatic response in the basal cells and a weak response correlating with glutamine independence of the luminal cells.

### Potential glutamine symbiosis between luminal and basal types of cells

**Figure 6:**
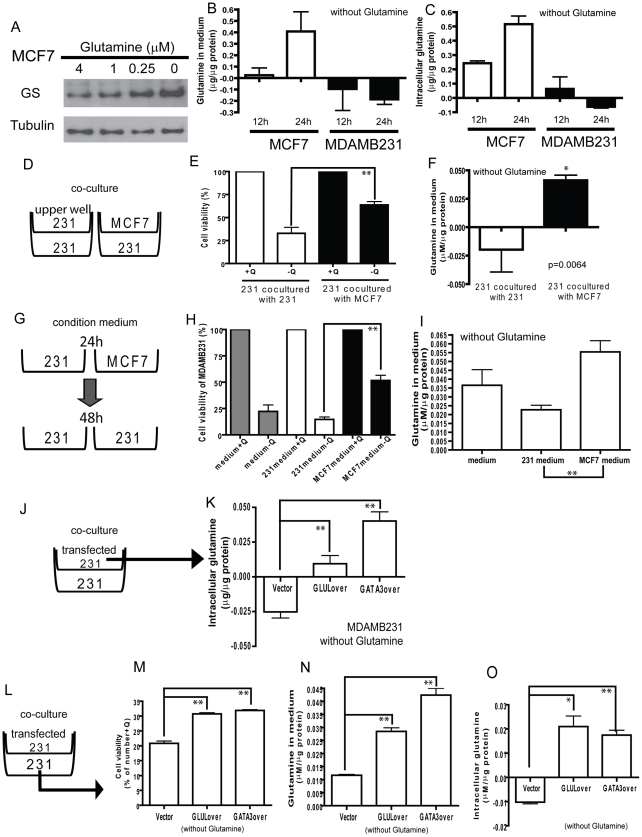
Potential for glutamine symbiosis between luminal and basal cells. (A) The protein levels of GS in MCF7 under different concentrations of glutamine. (B, C) The changes of the levels of glutamine in medium (B) and intracellular glutamine concentrations (C) in MCF7 and MDAMB231 cells deprived of glutamine for 12 and 24 h. (D) A diagram illustrating the co-culture systems in E, F. (E, F) The cell viability (E) and the glutamine levels in medium (F) when used to propagate MDAMB231 cells co-cultured with MDAMB231 or MCF7. (G) A diagram illustrating the condition medium model for H, I. (H, I) The cell viability (H) and the glutamine in medium (I) in MDAMB231 cells cultured with fresh medium, MDAMB231 or MCF7 condition medium. (J) A diagram illustrating the model of K. (K) The intracellular glutamine in vector transfected MDAMB231 cells. (L) A diagram illustrating the model of co-culture system for M-O. (M-O) The cell viability (M), the glutamine in medium (N), and intracellular glutamine concentrations (O) in MDAMB231 co-cultured with transfected MDAMB231 cells.

We examined how glutamine deprivation affected different glutamine-metabolizing enzymes and found that GS protein (Figure 6A), but not mRNA (Figure S7A), were significantly induced in MCF7 cells in a dosage-dependent manner. This translational regulation may be an adaptive response to compensate for reduced environmental levels of glutamine. To examine the role of GATA3 in the induction of GS during glutamine deprivation, we compared the GS protein levels under different glutamine levels in MCF-7 transfected with control or GATA3-targeting siRNA. We found that while the silencing of GATA3 reduced the GS levels, there was still significant protein induction during glutamine deprivation (Figure S7B). We also measured glutamine concentrations in glutamine deficient media used to culture MCF7 and MDAMB231 cells and found a significant increase in glutamine levels in medium cultured with MCF7 but not MDAMB231 cells (Figure 6B). Similarly, intracellular glutamine concentrations were increased only in MCF7 but not MDAMB231 cells under glutamine deprivation (Figure 6C). Therefore, the glutamine independence phenotype of luminal cells may be due to the capacity of these cells to synthesize glutamine from intracellular glutamate and other sources in the absence of external glutamine.

In normal breast ducts, luminal and basal cells are in close physical proximity. Because of the ability of luminal cells to synthesize glutamine and the requirement of basal cells for glutamine, we next tested the potential for glutamine symbiosis between these two cell types with transwell co-culturing experiments (Figure 6D–6F). We found that the viability of MDAMB231 cells under glutamine deficient media was significantly increased when MCF7 cells were used as a feeder layer (Figure 6E), consistent with observed higher extracellular glutamine levels (Figure 6F). Furthermore, conditioned medium from MCF7 cells was also able to support significantly the growth and viability of the MDAMB231 cells (Figure 6G–6I).

We showed above that increased levels of *GLUL* either by transfection with *GLUL* or *GATA3* makes the MDAMB231 line more resistant to glutamine deprivation (Figure 4C and 4H). We next asked whether this was due to increased synthesis of the nutrient. Intracellular glutamine levels increased dramatically in MDAMB231 cells expressing either *GLUL* or *GATA3* (5×10^4^ cells in the upper well) (Figure 6J, 6K). The effects of GLUL and GATA3 overexpression in MDAMB231 cells on intracellular glutamine levels were blocked with L-MS treatment (Figure S8A). We also showed that the intracellular glutamine levels were reduced in MCF7 with siRNAs targeted to GLUL or GATA3 in medium with normal glutamine level (Q4) or no glutamine (Q0) (Figure S8B). Further, in the co-culture system (Figure 6L), MDAMB231 cells demonstrated increased viability when co-cultured with either *GLUL* or *GATA3* expressing MDAMB231 cells (Figure 6M) and this correlated with both increased glutamine levels in the medium (Figure 6N) and intracellularly (Figure 6O). These data provide a consistent mechanistic picture of a gene expression program related to nutrient requirements and potential glutamine symbiosis.

### *GLUL* repression of *GLS* contributes to the cell type–specific expression of glutamine-metabolizing enzymes

**Figure 7:**
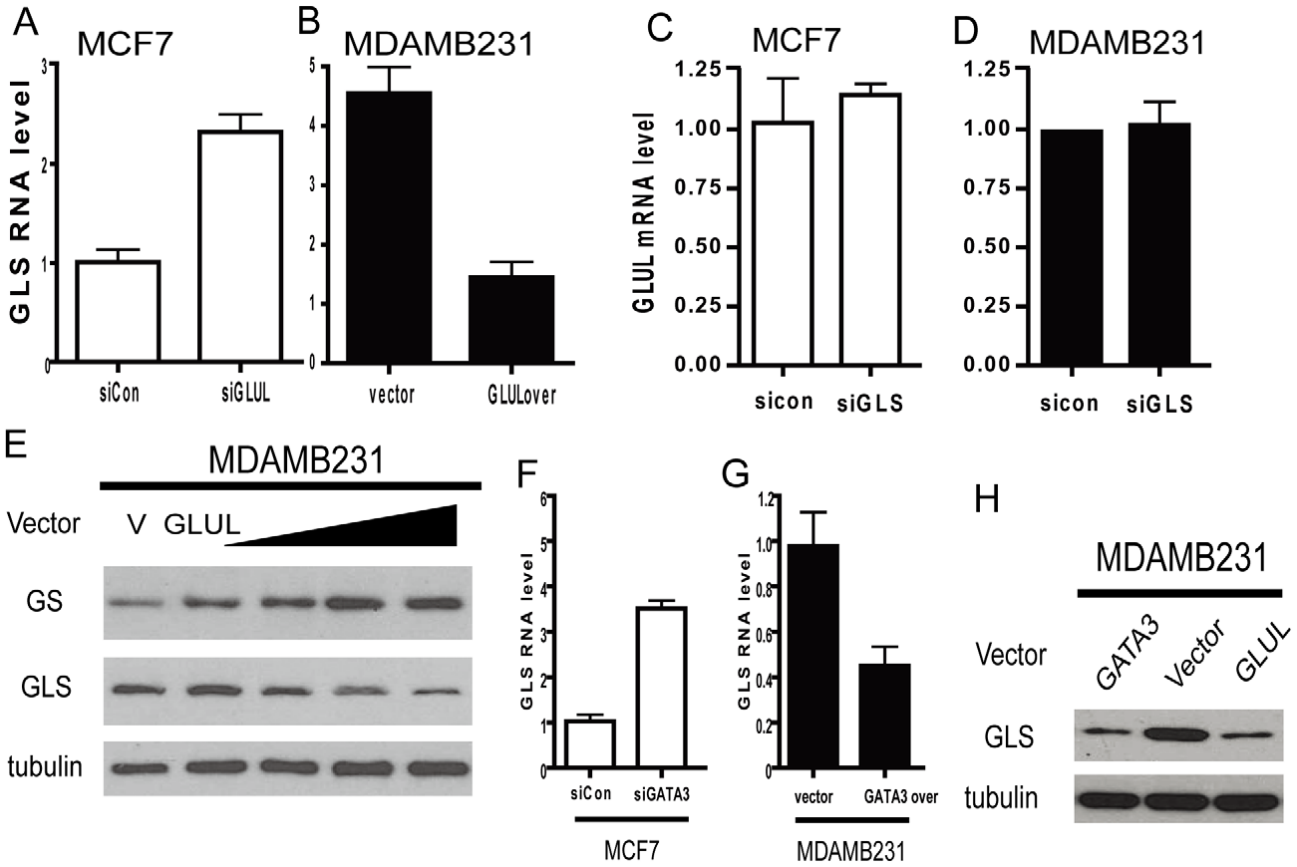
Repression of GLS by GLUL contributes to the polarized expression pattern. (A, B) The mRNA expression levels of *GLS* in the indicated MCF7 (luminal, empty) and MDAMB231 (basal, solid) when treated with indicated siRNAs or overexpression constructs. (C, D) The *GLUL* RNA expression levels in MCF7 (luminal, empty) and MDAMB231 (basal, solid) when treated with siRNA targeted to *GLS*. (E) *GLUL* and *GLS* protein expression levels in MDAMB231 treated with *GLUL* expression vector. (F, G) The expression of *GLS* mRNA in MCF7 (luminal, empty) and MDAMB231 (basal, solid) treated with indicated siRNAs or expression construct of GATA3. (H) The *GLS* protein expression levels in MDAMB231 cells with indicated expression constructs.

The expression of *GLUL* and *GLS* are inversely correlated in the luminal and basal types of primary breast cancers, cancer cell lines, and primary epithelial cells. This pattern of expression made us investigate whether cross-regulation exists between these two genes that encode enzymes mediating directly opposite chemical reactions. We first used siRNA to silence *GLUL* in MCF7 cells and observed an increase in *GLS* mRNA expression (Figure 7A). Further, ectopic over-expression of *GLUL* in MDAMB231 cells reduced *GLS* mRNA (Figure 7B). In contrast, similar silencing of *GLS* did not show any effect on *GLUL* levels (Figure 7C, 7D). The ability of *GLUL* overexpression in MDAMB231 to repress *GLS* was also seen at the protein level with a dose dependent decrease in *GLS* protein observed with increasing amounts of GS protein from varying levels of transfected GLUL (Figure 7E). These results indicated the ability of *GLUL* to repress the expression of *GLS* while *GLS* had no detectable effect on the level of *GLUL*.

Since GATA3 regulated the expression of *GLUL*, we tested the role of *GATA3* in regulating *GLS* by silencing and overexpressing *GATA3* in MCF7 and MDAMB231 cells, respectively. Silencing of *GATA3* in MCF7 cells increased *GLS* expression (Figure 7F) and *GATA3* overexpression in MDAMB231 significantly reduced the level of *GLS* (Figure 7G). These changes in *GLS* expression regulated by GATA3 were also detectable at the protein level compared with *GLUL* (Figure 7H). These results are also consistent with *GATA3* overexpression in the mouse epithelial cells (Figure 4D) [38].

### A proposed model for the regulation of glutamine dependence in breast cells

**Figure 8:**
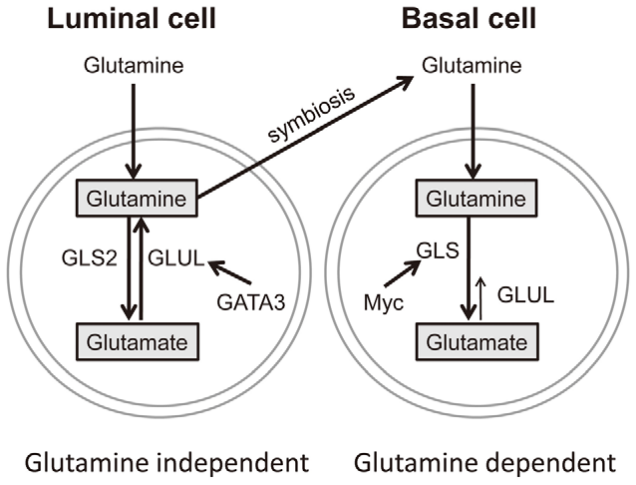
A model of glutamine metabolic regulation in different breast cells. The regulatory mechanisms of glutamine metabolic enzymes based on data from luminal and basal breast cells.

Based on the data presented, we propose that basal and luminal breast epithelial cells exhibit different patterns of glutamine metabolism (Figure 8). In the luminal cells, *GATA3* triggers expression of *GLUL* and contributes to glutamine independence. Furthermore, *GLUL* has the ability to repress *GLS* which would also help to maintain the cell-type specific expression pattern and phenotype. Basal-specific expression of *GLS* may be maintained by the absence of *GATA3* and higher activities of *c-myc* in the basal type cells [4], [46]. These findings suggest that glutamine deprivation may be a viable treatment strategy for basal-type breast cancers. In addition, the expression of *GLUL* in luminal type cancers correlates with the ability to synthesize glutamine from ammonia and glutamate, and therefore describes at the molecular level a type of cancer that is predicted to be more resistant to glutamine deprivation treatment.

## Discussion

While glutamine has been shown to be critical in many cancer types, its importance for breast cancers is not well defined. In this study, we found a cell lineage-specific variation in the response of basal and luminal breast cancer cells to glutamine deprivation. The basal-type breast cancer cells are dependent on glutamine and exhibit a phenotype of glutamine addiction. Such a phenotype of basal cells was previously reported to be sensitive to inhibitors of glutaminase [27], trans-amination by aspartate aminotransferase [47] and selective estrogen receptor modulators [48]. In contrast, the luminal-type breast cancer cells are much more glutamine-independent. We present a series of data which strongly suggest that this phenotypic difference is related to the luminal-specific expression of glutamine synthetase (GS encoded by the *GLUL* gene) which is in turn regulated by one of the key luminal transcription factors, *GATA3*. Further, GS itself represses the expression of glutaminase (*GLS*) to reinforce the metabolic pathway in the direction of glutamine synthesis in luminal breast cells and the potential for glutamine symbiosis with basal breast cells.

While variations in tumor metabolism can be caused by oncogenic events, our results highlight the importance of the differentiation status and cellular origins as a source of distinct metabolic patterns. Since differentiation state constitutes an important part of tumor heterogeneity, similar investigation into distinct metabolic needs may yield important information on how best to target tumor metabolism. As the induction of differentiation is an important component of some cancer therapeutics [49], such treatment-associated differentiation may also lead to changes in metabolic needs and may be exploited to enhance treatment efficacy. Similarly, the distinct nutrient requirements of tumor stem cells [50] may be used to target these unique populations which are more resistant to conventional cancer therapeutics.

### Genetic regulation of the cell type–specific expression of glutamine-metabolizing enzymes

The distinct glutamine requirement and differential expression of glutamine-metabolizing enzymes among luminal and basal breast cancers are consistent with our understanding of the genetic circuitry governing breast cancer subtypes and regulation of these glutamine-metabolizing enzymes. For example, the higher *GLS* level and sensitivity to glutamine deprivation of basal-type breast cancer cells are consistent with a high level of *c-myc* activity in basal cells [51], [52] and the recently described role of *c-myc* in regulating *GLS* [24], [25]. The higher levels of *GLS* are also consistent with the susceptibility to growth inhibition by targeting this enzyme [27] and indicate the essential nature of this metabolic pathway in the basal cells. These results indicate that triple-negative basal-like breast tumors, with few current therapeutic options, are addicted to glutamine and may benefit from glutamine-targeting therapies [22], [27]. In contrast, the luminal specific expression of *GLS2* may reflect the higher p53 (wild type) activity in luminal cells [4], [52] given the ability of *p53* to regulate *GLS2* [53], [54]. Our results suggest that GATA3 is directly involved in the transcriptional regulation of *GLUL* in luminal cells.

### The spatial and cell-type expression of glutamine synthetase and glutaminase

The spatial and cell type specific expression of *GLUL* (GS) seen in our studies on breast epithelial cells is also observed in several other cellular contexts. In the brain, GS is expressed mainly in glial cells [55] and is responsible for the synthesis of glutamine from the uptake of glutamate secreted by adjacent neurons. Similar spatial division of glutamine degradation and synthesis also occurs in distinct patterns of GS and GLS expression in the liver [56]. Glutamine degradation by GLS occurs in the periportal cells where there is a high glutamine level from the digested nutrients in the gastrointestinal tract. In contrast, the expression of *GLUL* (GS) is restricted to zones of hepatocytes surrounding the central lobular vein with lower glutamine levels [56]. In the renal nephron, *GLUL* (GS) expression is restricted to the straight portion of the proximal tubules and plays an important role in the removal of ammonia [57]. Such physical separation of glutaminase and glutamine synthetase associated with differentiation and nutrient availability coordinate the glutamine synthesis and effective detoxification of ammonia and glutamate. Similar distinct glutamine metabolism in luminal and basal breast epithelial cells also appears to impact tumors derived from these different lineages and opens an additional window into the metabolic phenotypes of this heterogeneous disease. Therapeutic interventions based upon metabolic targets will need to incorporate these systematic differences between tumor subtypes.

### Inter-cellular metabolic symbiosis

Under glutamine deprivation, the high mRNA levels of *GLUL* (GS) in luminal cells undergo further protein upregulation to provide glutamine and may also support the glutamine requirement in basal cells in physical proximity. Similar nutritional and metabolic interaction underlies many symbiotic relationships among different organisms and cell types, including the symbiotic nitrogen-fixing root nodules on legumes [58], the mutualistic symbiosis between bacteria and insects [59], and the glutamate-glutamine shuttle between neurons and astroglial cells in the brain [55], [60]. Interestingly, GS plays a critical role in all these diverse examples of metabolic symbiosis. In addition to the inter-cellular exchange of nutrients, the synthesis of glutamine from glutamate and ammonia by GS can also remove the potential toxicity from the accumulation of glutamate (neurotransmitter) and ammonia (nitrogen waste). Ammonia from glutaminolysis has been shown to act as a diffusible autocrine- and paracrine substance inducing autophagy [61]. Given the physical proximity between basal and luminal cells in breast ducts, such a reciprocal metabolic relationship may also be relevant under different environmental or growth conditions. When the tissue organization is disrupted in malignancy, glutamine dependence of the basal-type tumors may be exploited to treat this type of aggressive cancers.

### The therapeutic implications

Our findings strongly suggest that there will be significant variation in response to glutamine-targeting therapies. Among breast cancers, systematic variation in the glutamine consumptive vs. synthetic behaviors seen in the basal and luminal tumors will directly influence this response. Similar heterogeneity may be important in other tumor types as well. Our data also provide evidence that glutamine-targeting therapeutics may be of special clinical utility for the triple-negative basal-like breast tumors with few therapeutic options. Many current glutamine-targeting therapeutics focus on glutaminase [25], [27], but the cell-type specific expression of *GLS* and *GLS2* in different tumors indicates the importance of choosing compounds with intended specificity for particular glutaminase activities in the treated tumors. Since GS is a key genetic determinant of glutamine independence in luminal cells, the evaluation of the GS levels in tumors may be helpful in predicting response. In addition to the cell-autonomous variations in the GS expression and response to glutamine deprivation, the efficacy of glutamine-targeting therapies may also be affected by the ability of adjacent non-transformed cells to provide glutamine. It is important to note that GS activities have been reported in fibroblasts [62] and macrophages [63]. The availability of glutamine from other non-tumor cells or blood may reduce the efficacy of glutamine-targeting therapies. Thus, GS inhibition may be combined with glutamine-targeting therapies to further enhance efficacy and reduce resistance, similar to the use of GS inhibitors to sensitize cancer cells to L-asparaginase [64].

With the explosion of genomic data, we have obtained significant knowledge on how genetic dysregulation contributes to tumor heterogeneity in human cancers. Since dysregulated metabolism is an essential part of oncogenesis, similarly detailed knowledge of metabolic profiles may be of equal or greater importance in understanding and treating the disease [65], [66].

## Materials and Methods

### Cell culture

All breast cancer cells were cultured in DMEM with 4.5 g/L glucose, supplemented with 10% fetal bovine serum and 1% penicillin/streptomycin in 5% CO2.

Primary luminal and basal cells were obtained from women undergoing breast reduction for non-malignant conditions and were separated by cell surface binding to the TACSD1 protein of the Ber-Ep4 antibody as described [12].

### Cell viability assays

For the MTT assay, 2.5×10^3^ cells in 100 µl of medium were seeded in a 96-well culture plate. After treatments, cell number was evaluated. In brief, 10 µl of MTT (Sigma M5655) (0.5 mg/ml) was added to each well, and then the plates were incubated at 37°C for 3 h. The formazan product was dissolved in DMSO, and the absorbance at 570 nm was measured using a microplate reader. To measure viability by direct counting, 2×10^4^ cells were seeded in 12-well dishes and treated with medium containing different concentrations of glutamine for 48 h; the cells were collected and stained with 0.4% Trypan Blue. Cells excluding and taking up dye were counted on a hemocytometer under phase contrast microscopy. For glutamine synthetase inhibition, L-MS (L-Methionine-Sulfoximine, 5 mM, Sigma-Aldrich) was administered to cells for 48 h.

### ATP level measurement

Cells (5×10^3^/well of a 96 well dish) were treated with or without glutamine for 12 h and ATP content was measured in accordance with the protocol of the ATP-Lite luminescent ATP detection assay kit (Perkin-Elmer). Briefly, 100 µl of assay reagent was added to the wells and mixed for 10 min in the dark; intracellular ATP content was measured using a luminescence multi-label counter. The ATP levels were normalized based on cell counts measured by the MTT assay.

### Determination of glutamine concentration

Cells (1×10^4^/well) in a 24 well plate were cultured for 24 h in medium without phenol-red, medium was collected, and cells were lysed with RIPA buffer (Sigma-Aldrich). Concentrations of glutamine in the medium and in the cell lysate were determined with the glutamine/glutamate determination kit (GLN-1; Sigma-Aldrich). Each sample was divided into two parts; part 1 was measured with glutaminase for transferring the glutamine into glutamate, part 2 was measured directly. Samples were then dehydrogenized to α-ketoglutarate accompanied by reduction of NAD+ to NADH. The amount of NADH is proportional to the amount of glutamate and was measured using a spectrophotometer at 340 nm. A standard curve was determined for each experiment to calculate the concentration of glutamate in samples. Glutamine levels were calculated (part 1 minus part 2) and normalized to total protein levels. The glutamine level of normal culture medium was also measured, and the glutamine consumption was calculated as (glutamine in normal medium-glutamine in medium after culturing cells) and normalized to protein level.

### Western blot analyses

Proteins were separated by 10–12% SDS–PAGE and transferred to Immobilon-P membranes (Millipore). Membranes were blocked with 5% skim milk, incubated with primary antibodies (GLUL, G2781, Sigma; GLS, ab60709, Abcam; GATA3, sc269, Santa Cruz; GLS2, ab91073, Abcam; tubulin, 2128, cell signaling), HRP-conjugated secondary antibody (Perkin-Elmer), and detected with the ECL Western blotting reagent (Amersham).

### Microarray analysis

MCF7 and MDAMB231 cells were cultured in medium with or without glutamine for 24 h in triplicate. RNAs were collected with MirVana kit (Ambion) and hybridized to Affymetrix U133A2 arrays. Probe intensities were normalized by RMA and then the changes of expression by glutamine deprivation (0 mM glutamine/Q0) were derived by zero-transformation against the corresponding cells grown in glutamine containing medium (4 mM glutamine/Q4).

### RNA interference and overexpression

Cells were transfected with non-targeting control or synthetic siRNAs targeting *GLUL*, *GLS*, *GLS2* and *GATA3* (Applied Biosystems) with lipofectamine 2000 (Invitrogen). For overexpression experiments, empty vector or overexpression constructs for *GLUL* or *GATA3* (Origene) were transfected into cells with lipofectamine 2000 for 48 hours before the levels of indicated transcripts and proteins were examined by real-time RT-PCR and western blot.

### Real-time RT-PCR

Total RNA was reverse-transcribed to cDNA with SuperScript II reverse transcription kit, then used for real-time PCR with Power SYBR Green PCR Mix (Applied Biosystems) and indicated primers (Table S1), and normalized to β-actin mRNA levels measured in parallel.

### ChIP assay

10% formaldehyde solution was added to cells to crosslink DNA-protein complexes. Isolated nuclear chromatin extracts were sonicated and incubated overnight at 4°C with either anti-GATA3 (SC269, Santa Cruz) or normal mouse IgG (SC3878, Santa Cruz). This was followed by incubation with 20 ml of Protein G agarose beads (Roche) for 4 hours at 4°C. After extensive washing, DNA fragments were harvested by de-crosslinking the immunoprecipitates. Real time-PCR utilizing SYBR Green master mix (Applied Biosystems) was performed to check the enrichment of indicated promoter regions in pull-down samples using primers listed in Table S1 and normalized with albumin.

### Co-culture and conditioned medium

For co-culture experiments, MDAMB231, MCF7, or transfected MDAMB231 cells were seeded in minicells (upper well/5×10^4^ cells) with 0.4 µm pores (Millipore) and co-cultured with MDAMB231 (lower well/1×10^4^ cells) for 12, 24 or 48 h in 24-well plates. For conditioned medium experiments, MDAMB231 or MCF7 cells (5×10^4^) were seeded in a 24-well plate and incubated in medium with or without glutamine for 24 hours and then medium was transferred to new wells containing MDAMB231 cells (1×10^4^). In 12 and 24 hours experiments, medium was collected; cells were washed by PBS and then lysed with 100 µl RIPA buffer. Glutamine concentration was measured with GLN-1 (Sigma). In 48 h experiments, cell numbers were counted by trypan blue exclusion assay.

### Statistics

All experiments were expressed as mean ± standard deviation (SD) with t-test. Statistical significance was calculated by t test, considering p<0.05 (*) and p<0.01(**) as statistically significant.

## Supporting Information

Figure S1 Comparison of expression levels of *GLUL* (A), *GLS* (B), and *GLS2* (C) in two breast tumors datasets with 5 intrinsic subtypes in the breast tumor dataset (Chin et al., 2006). **: p<0.01.
(TIF)

Figure S2 The RNA and protein levels of GLUL in MCF7 which has been transfected with non-target control or siRNAs targeting *GLUL*. (A, B) The RNA (A) and protein (B) levels were measured by real time-PCR and western blotting in MCF7 cells treated with indicated siRNAs.
(TIF)

Figure S3 The normalized cell viability of MCF7 transfected with siRNAs targeted to *GLUL*, *GATA3*, or *GLS2* culturing in medium with or without glutamine for 48h.
(TIF)

Figure S4 The RNA and protein levels of *GLUL* in MDAMB231 cells which has been transfected with overexpression construct of *GLUL*. (A, B) The RNA (A) and protein (B) levels of GLUL were measured in MDAMB231 with GLUL overexpression.
(TIF)

Figure S5 The RNA and protein levels of *GATA3* and *GLUL* in cells which have been transfected with indicated siRNAs or indicated overexpression constructs. (A) The mRNA level of *GATA3* in the MCF-7 and MDAMB231 cells. (B, C) The RNA (B) and protein (C) levels of *GATA3* in MCF7 which has been transfected with siRNAs targeting *GATA3*. (C) The protein expression of GS in MCF7 transfected with siRNA targeting *GATA3*. (D) The RNA level of *GATA3* in MDAMB231 cells transfected with control or *GATA3* overexpression constructs. (E) The protein expression of GS in MDAMB231 with GATA3 overexpression.
(TIF)

Figure S6 L-MS partially abolished the effects of *GLUL* or *GATA3* overexpression in cell survival in MDAMB231 cells. (A, B) The effect of L-MS on cell survival of MDAMB231 cells transfected with empty vector, *GLUL* (A), and *GATA3* (B) under normal (Q4) or no glutamine (Q0) conditions for 24h.
(TIF)

Figure S7 The RNA and protein expressions of GLUL. (A) The RNA expressions of *GLUL* in MCF7, (B) The protein levels of GS in 24h under different dose of glutamine deprivations in MCF7 cells transfected with control or siRNA targeting *GATA3*.
(TIF)

Figure S8 The intracellular glutamine levels in MDAMB231 and MCF7 cells. (A) The intracellular glutamine concentrations in MDAMB231 cells which have been transfected with vector, *GLUL* and *GATA3*, in combination with or without L-MS under glutamine deprivation in 24h. (B) The intracellular glutamine levels in MCF7 with siRNA of non-target control (siCON), *GLU*L (siGLUL) or *GATA3* (siGATA3) under normal (Q4) or no glutamine (Q0) medium.
(TIF)

Table S1 The sequences of all primers used in real time-PCR.
(DOC)
