# Supplementary figures and images for: Glutamine Synthetase Is a Genetic Determinant of Cell Type–Specific Glutamine Independence in Breast Epithelia
Source: PLoS Genet. 2011 Aug 11;7(8):e1002229. doi: 10.1371/journal.pgen.1002229 (PMC3154963; doi:10.1371/journal.pgen.1002229)

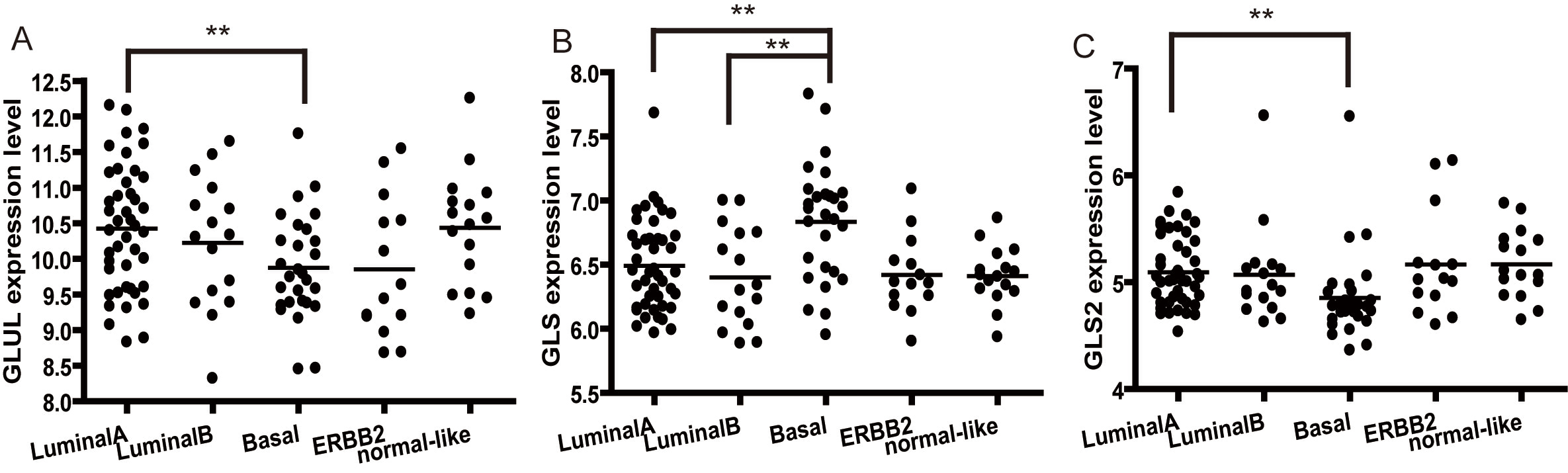

Supplement: Figure S1 — Comparison of expression levels of GLUL (A), GLS (B), and GLS2 (C) in two breast tumors datasets with 5 intrinsic subtypes in the breast tumor dataset (Chin et al., 2006). **: p<0.01. (TIF) [file pgen.1002229.s001.tif]

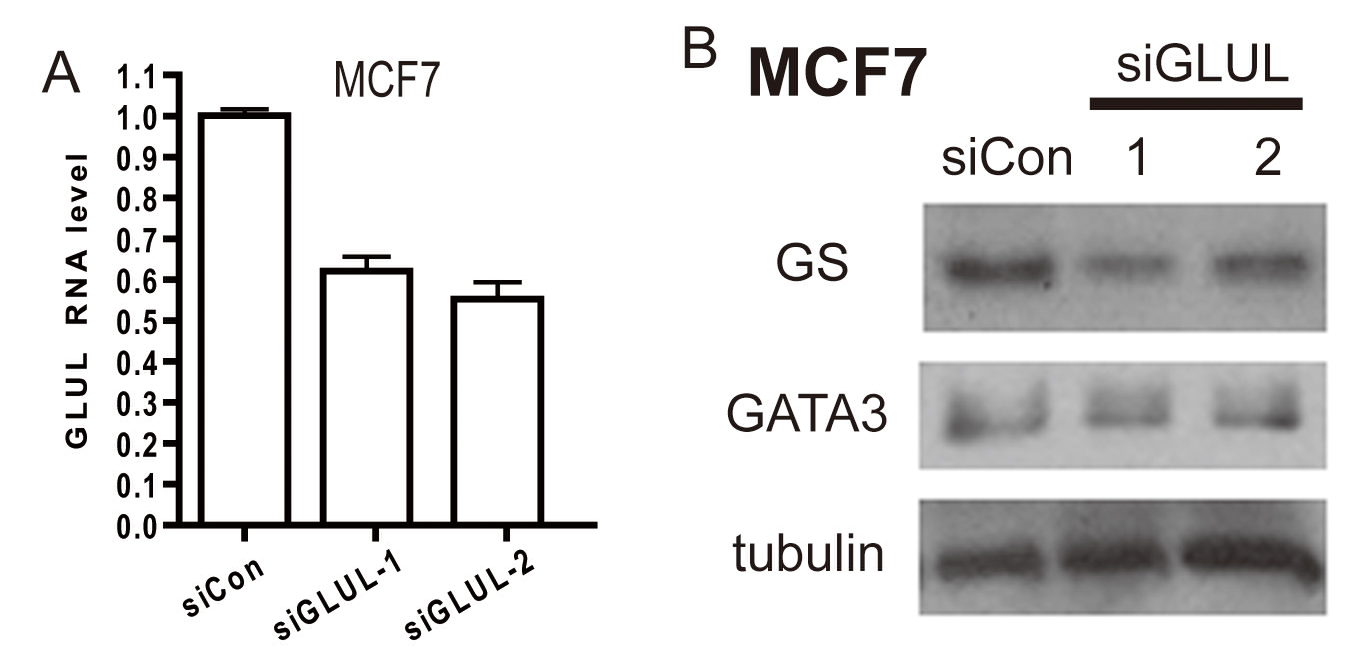

Supplement: Figure S2 — The RNA and protein levels of GLUL in MCF7 which has been transfected with non-target control or siRNAs targeting GLUL. (A, B) The RNA (A) and protein (B) levels were measured by real time-PCR and western blotting in MCF7 cells treated with indicated siRNAs. (TIF) [file pgen.1002229.s002.tif]

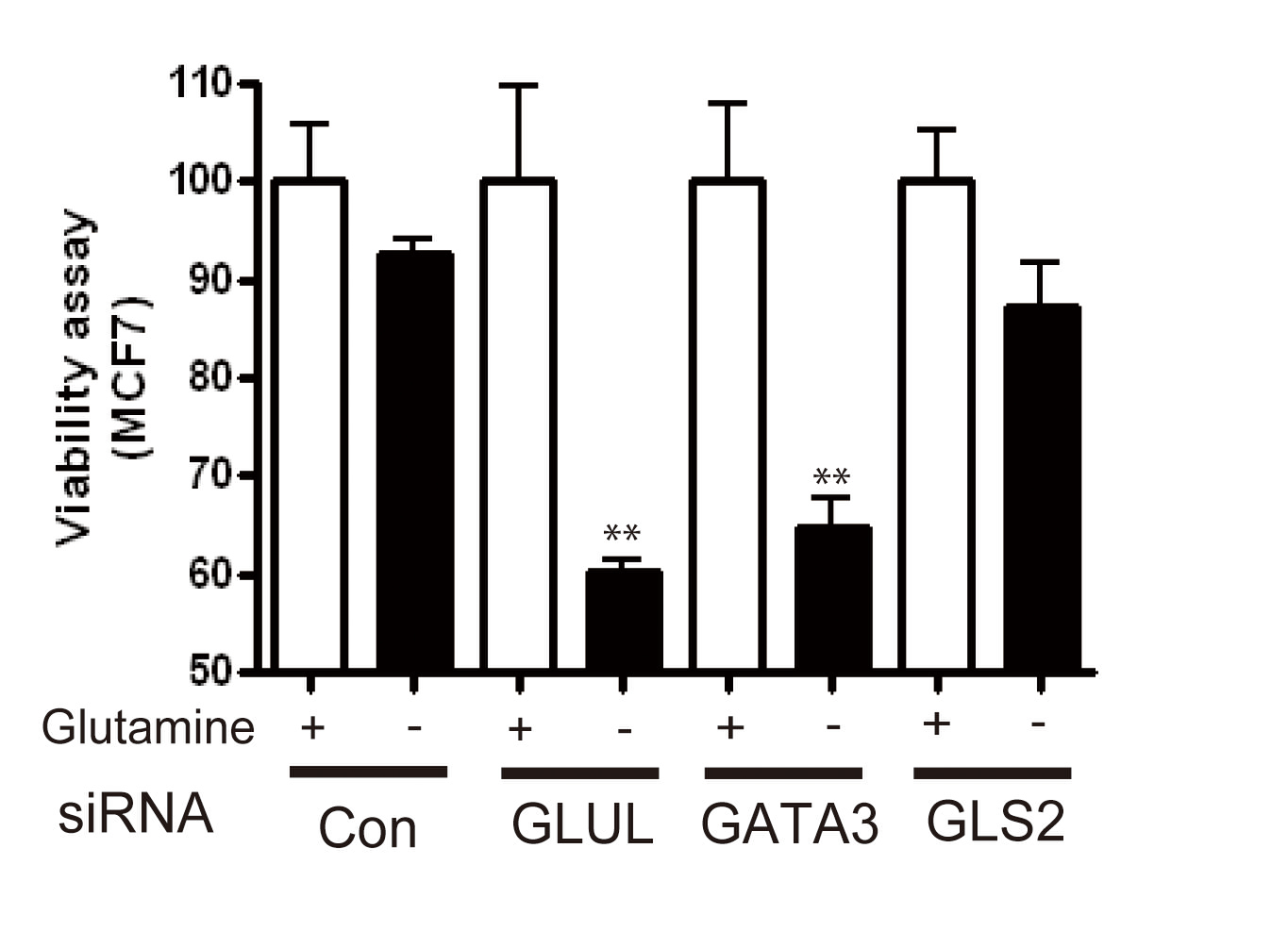

Supplement: Figure S3 — The normalized cell viability of MCF7 transfected with siRNAs targeted to GLUL, GATA3, or GLS2 culturing in medium with or without glutamine for 48h. (TIF) [file pgen.1002229.s003.tif]

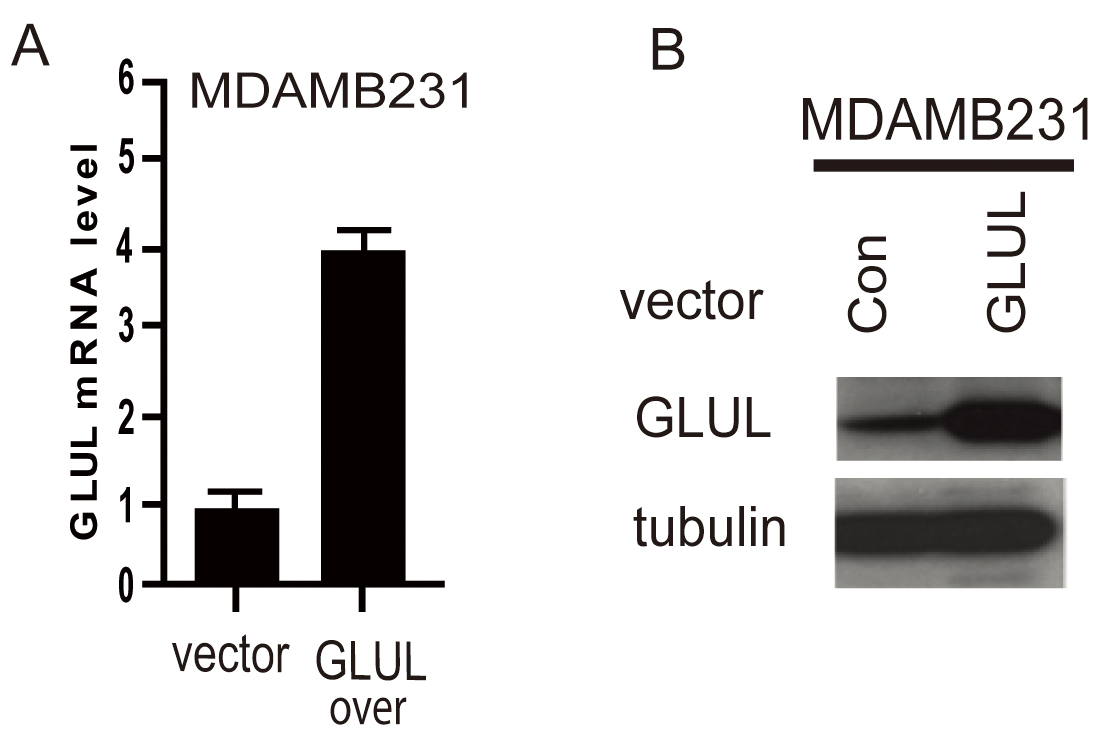

Supplement: Figure S4 — The RNA and protein levels of GLUL in MDAMB231 cells which has been transfected with overexpression construct of GLUL. (A, B) The RNA (A) and protein (B) levels of GLUL were measured in MDAMB231 with GLUL overexpression. (TIF) [file pgen.1002229.s004.tif]

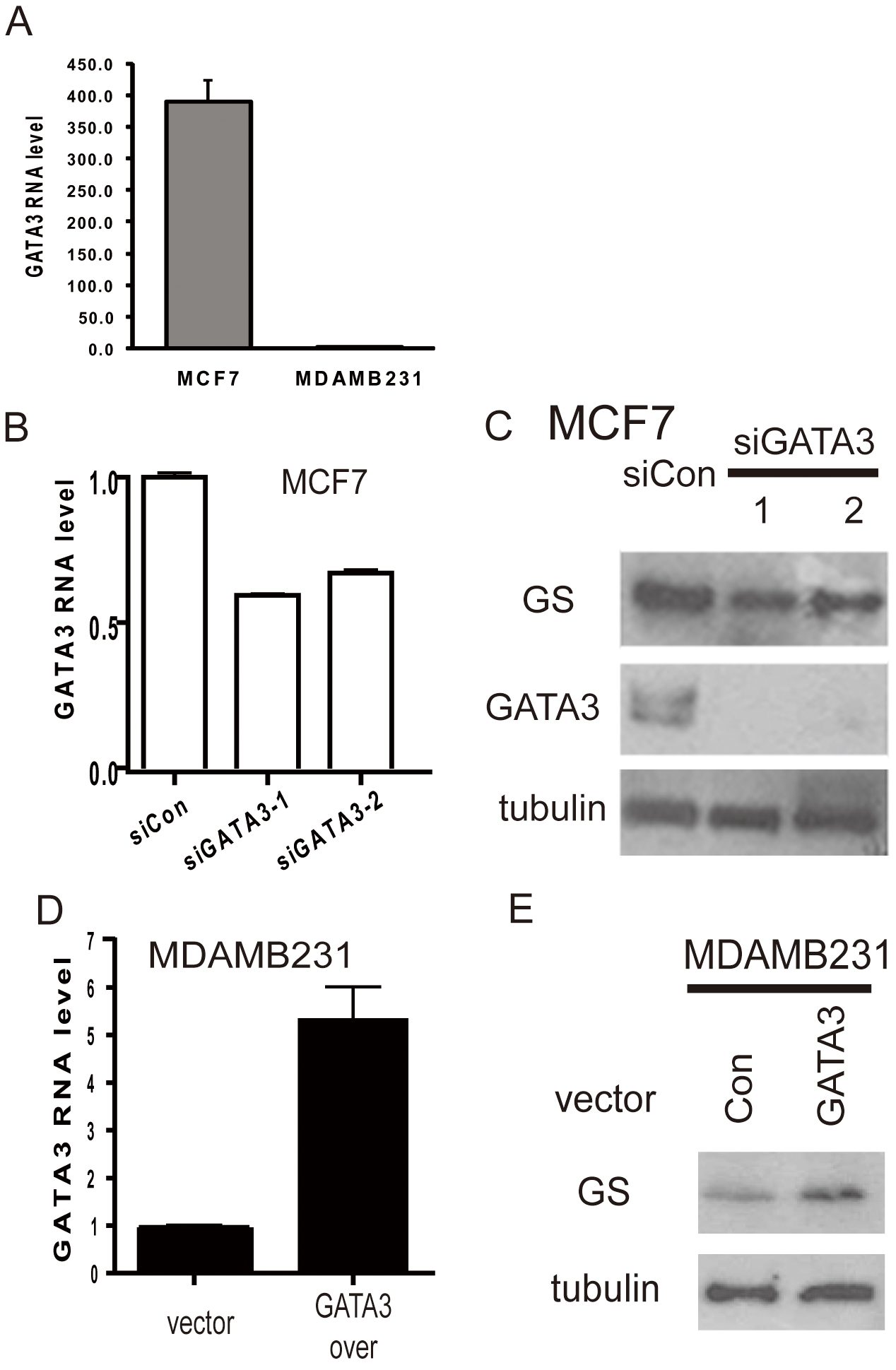

Supplement: Figure S5 — The RNA and protein levels of GATA3 and GLUL in cells which have been transfected with indicated siRNAs or indicated overexpression constructs. (A) The mRNA level of GATA3 in the MCF-7 and MDAMB231 cells. (B, C) The RNA (B) and protein (C) levels of GATA3 in MCF7 which has been transfected with siRNAs targeting GATA3. (C) The protein expression of GS in MCF7 transfected with siRNA targeting GATA3. (D) The RNA level of GATA3 in MDAMB231 cells transfected with control or GATA3 overexpression constructs. (E) The protein expression of GS in MDAMB231 with GATA3 overexpression. (TIF) [file pgen.1002229.s005.tif]

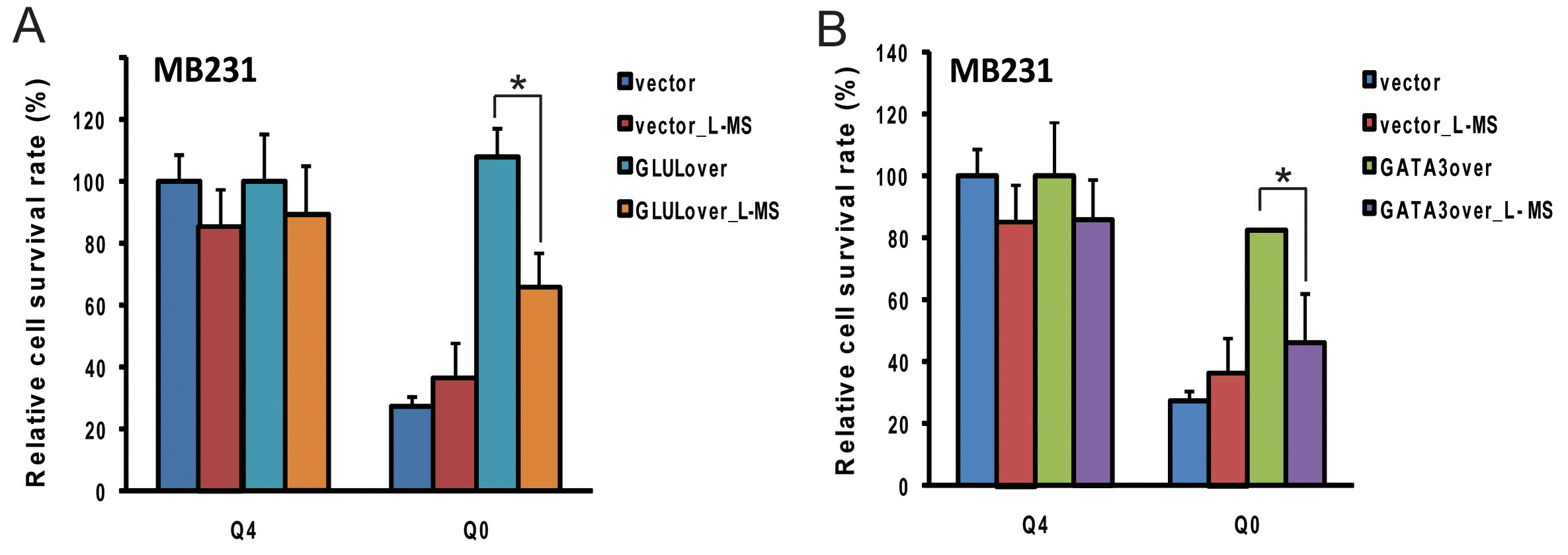

Supplement: Figure S6 — L-MS partially abolished the effects of GLUL or GATA3 overexpression in cell survival in MDAMB231 cells. (A, B) The effect of L-MS on cell survival of MDAMB231 cells transfected with empty vector, GLUL (A), and GATA3 (B) under normal (Q4) or no glutamine (Q0) conditions for 24h. (TIF) [file pgen.1002229.s006.tif]

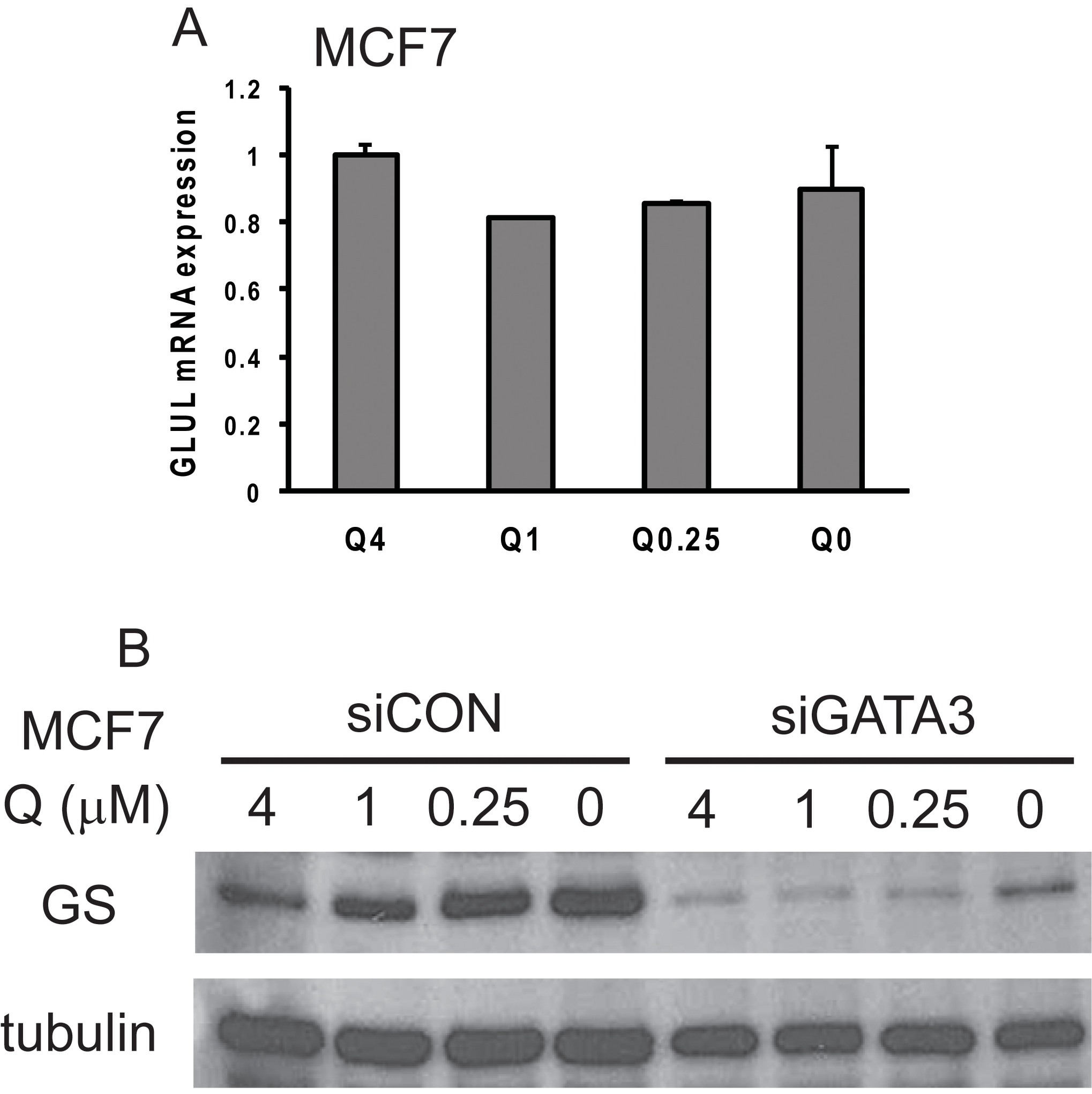

Supplement: Figure S7 — The RNA and protein expressions of GLUL. (A) The RNA expressions of GLUL in MCF7, (B) The protein levels of GS in 24h under different dose of glutamine deprivations in MCF7 cells transfected with control or siRNA targeting GATA3. (TIF) [file pgen.1002229.s007.tif]

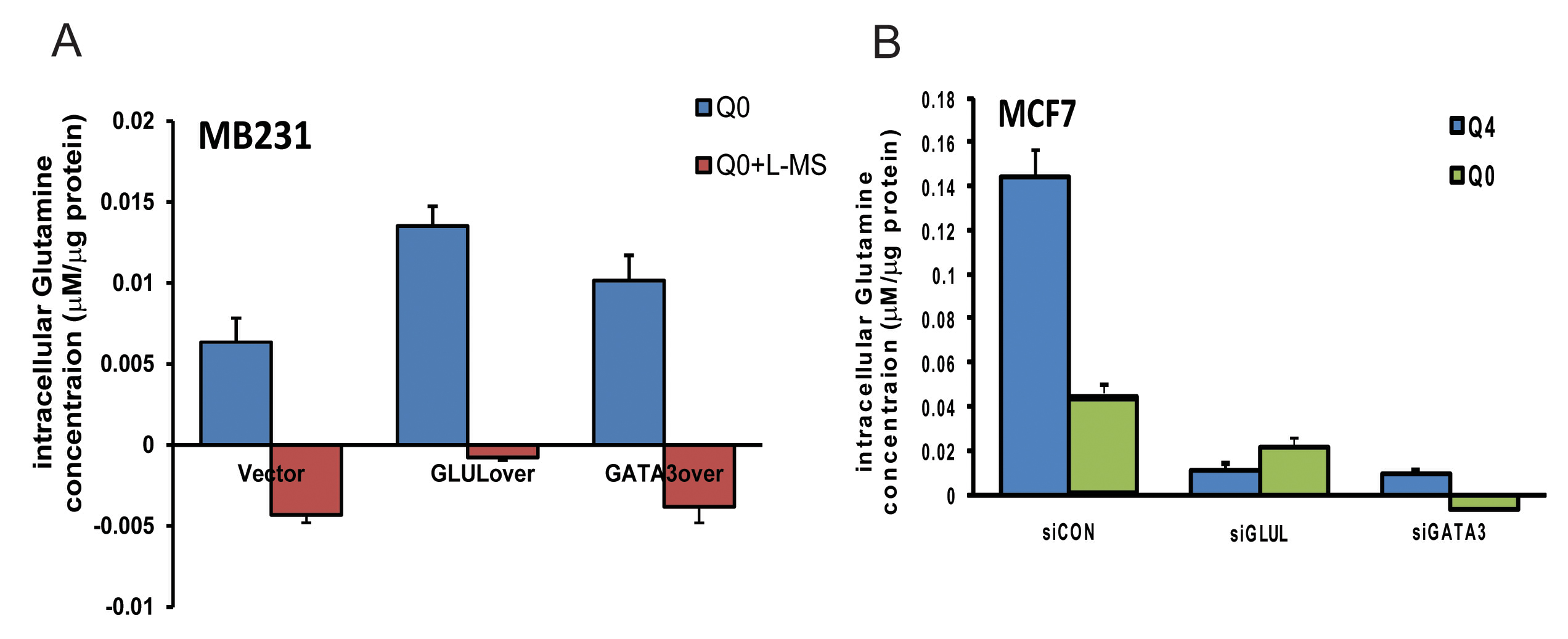

Supplement: Figure S8 — The intracellular glutamine levels in MDAMB231 and MCF7 cells. (A) The intracellular glutamine concentrations in MDAMB231 cells which have been transfected with vector, GLUL and GATA3, in combination with or without L-MS under glutamine deprivation in 24h. (B) The intracellular glutamine levels in MCF7 with siRNA of non-target control (siCON), GLUL (siGLUL) or GATA3 (siGATA3) under normal (Q4) or no glutamine (Q0) medium. (TIF) [file pgen.1002229.s008.tif]
