# Supplementary material for: Glutamine Synthetase Is a Genetic Determinant of Cell Type–Specific Glutamine Independence in Breast Epithelia
Source: PLoS Genet. 2011 Aug 11;7(8):e1002229. doi: 10.1371/journal.pgen.1002229 (PMC3154963; doi:10.1371/journal.pgen.1002229)
Supplement: Table S1 — The sequences of all primers used in real time-PCR. (DOC) [file pgen.1002229.s009.doc]

**Supplemental Table1:**

**Realtime-PCR primers**:

|  | |  |
| --- | --- | --- |
| Actin | forward | CTCTTCCAGCCTTCCTTCCT |
|  | reverse | AGCACTGTGTTGGCGTACAG |
| GLUL | forward | CCTGCTTGTATGCTGGAGTC |
|  | reverse | GATCTCCCATGCTGATTCCT |
| GLS | forward | GCTGTGCTCCATTGAAGTGA |
|  | reverse | GCAAACTGCCCTGAGAAGTC |
| GLS2 | forward | ATCAGAAAGTGGCATGCTGT |
|  | reverse | GCCTTTAGTGCAGTGGTGAA |
| GATA3 | forward | GCGGGCTCTATCACAAAATGA |
|  | reverse | GCTCTCCTGGCTGCAGACAGC |
| XBP1 | forward | AGTCCGCAGCACTCAGACTA |
|  | reverse | GGGTCCAAGTTGTCCAGAAT |
| DDIT3 | forward | ATGAACGGCTCAAGCAGGAA |
|  | reverse | GCAGATTCACCATTCGGTCAA |
|  |  |  |
| Real time primers used in ChIP assay | | |
| ESR1 | forward | TGTAGGCTAGTTTTGTTTAACGATTTTT |
|  | reverse | GGTGATGGGAGAATTGCTTAGAA |
| GLUL-A | forward | CGTGCTGGTGCATTTTCTGT |
|  | reverse | ATTCTCTTCCCCCAGTGGC |
| GLUL-B | forward | TTTACGTGCTCACCAAAGTG |
|  | reverse | TGAAGCCAATTCTTGAAAGC |
| Albumin | forward | TACATTGACAAGGTCTTGTGGAG |
|  | reverse | TGGGGTTGACAGAAGAGAAAAGC |
